# Highly Specific Antibodies for Co-Detection of Human Choline Kinase α1 and α2 Isoforms

**DOI:** 10.1371/journal.pone.0012999

**Published:** 2010-09-27

**Authors:** Wei Cun See Too, Mun Teng Wong, Ling Ling Few, Manfred Konrad

**Affiliations:** 1 School of Health Sciences, Universiti Sains Malaysia, Kubang Kerian, Kelantan, Malaysia; 2 Max Planck Institute for Biophysical Chemistry, Goettingen, Germany; Indiana University, United States of America

## Abstract

**Background:**

Choline kinase is the first enzyme in the CDP-choline pathway that synthesizes phosphatidylcholine, the major phospholipid in eukaryotic cell membranes. In humans, choline kinase exists as three isoforms (CKα1, α2, and β). Specific inhibition of CKα has been reported to selectively kill tumoral cells. Monoclonal and polyclonal antibodies against CKα used in previous studies to detect the level of this isozyme in different cellular or biochemical contexts were able to detect either the α1 or the α2 isoform.

**Methodology/Principal Findings:**

In this study, an antiserum against CKα was produced by immunizing rabbits with denatured, purified recombinant CKα2 full-length protein. This antiserum was highly specific for CKα when tested with extracts from different cell lines, and there was no cross reactivity with purified CKβ and other related proteins like human ethanolamine kinases (EK) and yeast choline or ethanolamine kinases. The antiserum simultaneously detected both CKα1 and α2 isoforms in MCF-7 and HepG2 cell extracts, but not in HeLa, HCT-116, and mouse embryonic stem cell extracts. Subsequent protein dot blot assay of total CKα in a human normal/tumor protein array of 30 tissue samples by using the antiserum showed that CKα was not overexpressed in all tumor tissues when compared to their normal counterparts. Most striking differences between tumor and normal CKα expression levels were observed in kidney (11-fold higher in tumor) and liver (15-fold lower in tumor) samples.

**Conclusion/Significance:**

Apart from its high sensitivity and specificity, the antiserum produced in this work, which does not require further purification, has the advantage of co-detecting both α1 and α2 isoforms in cell extracts for direct comparison of their expression levels.

## Introduction

Choline kinase (CK) (EC 2.7.1.32) catalyzes the phosphorylation of choline by ATP in the presence of Mg^2+^, yielding phosphocholine and ADP [1]. CK commits choline to the so-called Kennedy pathway for the biosynthesis of phosphatidylcholine (PtdCho) [2]. PtdCho is the predominant membrane lipid in eukaryotes amounting to almost 50% of the total phospholipid content [3]. Apart from its traditional role in the biosynthesis of PtdCho, studies in the last decade have linked CK with muscular dystrophy, bone deformities and cancer [4]. In mammals including humans, CK exists as at least three isoforms, encoded by two separate genes named *ck-α* and *ck-β*. While *ck-β* codes for a single protein (CKβ), *ck-α* undergoes alternative splicing and is thus responsible for the production of two CK isoforms, α1 and α2 [1] which differ only by the presence of an additional stretch of 18 amino acids present in the α2 isoform [5] encoded by exon 3 of the α2 transcript.

Increased CK activity was found in human breast cancer, and overexpression of CK is frequently observed in lung, prostate and colorectal cancers [6]. Elevated levels of CK activity in response to treatment of rats with xenobiotics such as aromatic hydrocarbons [1], or treatment of cultured cells with growth stimulants such as serum, epidermal growth factors, or insulin [7], [8], has been reported. Recently, it was shown that overexpression of CK increased the invasiveness and drug resistance of MCF-7 human breast cancer cells [9]. Furthermore, CK suppression by RNA interference in breast cancer cells reduced proliferation and induced differentiation [10]. Based on these observations, CK inhibition has been proposed as a potential anticancer strategy [11], [12], [13]. More recent studies on the biological function of CK isozymes revealed that CKα may play a more prominent role in cancer development as compared to CKβ, as only CKα was upregulated in breast cancer cell lines [14], and specific depletion of the CKα isoform by shRNA selectively induced apoptosis in several tumor-derived cell lines without affecting the viability of normal primary cells [15]. The CKα isoform has also been proposed as a new prognostic marker for predicting the clinical outcome in patients with non-small-cell lung cancer [16].

Immunoblot detection of CKα has become the primary means to evaluate the level of this isoform in various normal and cancer cell lines as well as upon treatments such as RNA interference. Polyclonal [17] and monoclonal [18] antibodies that specifically recognize CKα have previously been developed. Commercial polyclonal anti-CKα antibody has been used to assess the level of this isoform in CKα knockout mice [19]. However, all the antibodies used detected the expression of either CKα1 or α2 in the same sample. The expression of CKα was generally referred to a single band in Western detections and no attempt has been made to investigate the differential expression of α1 and α2 in various experimental settings.

In this work, a polyclonal antibody against human CKα was generated in rabbits using recombinantly produced CKα2 as antigen. The antiserum was tested for cross-reactivity with purified human and yeast choline and ethanolamine kinase isoforms, and the sensitivity was assessed by detecting variable amounts of purified CKα2. The specificity of the antiserum was confirmed by immunoblot detection of CKα in different cancer cell lines. Subsequently, the antiserum was used in protein dot blot assays to determine the level of total CKα and analyze its presence in 15 pairs of normal and tumor tissues.

## Results

### High sensitivity and specificity of CKα antiserum

**Figure 1:**
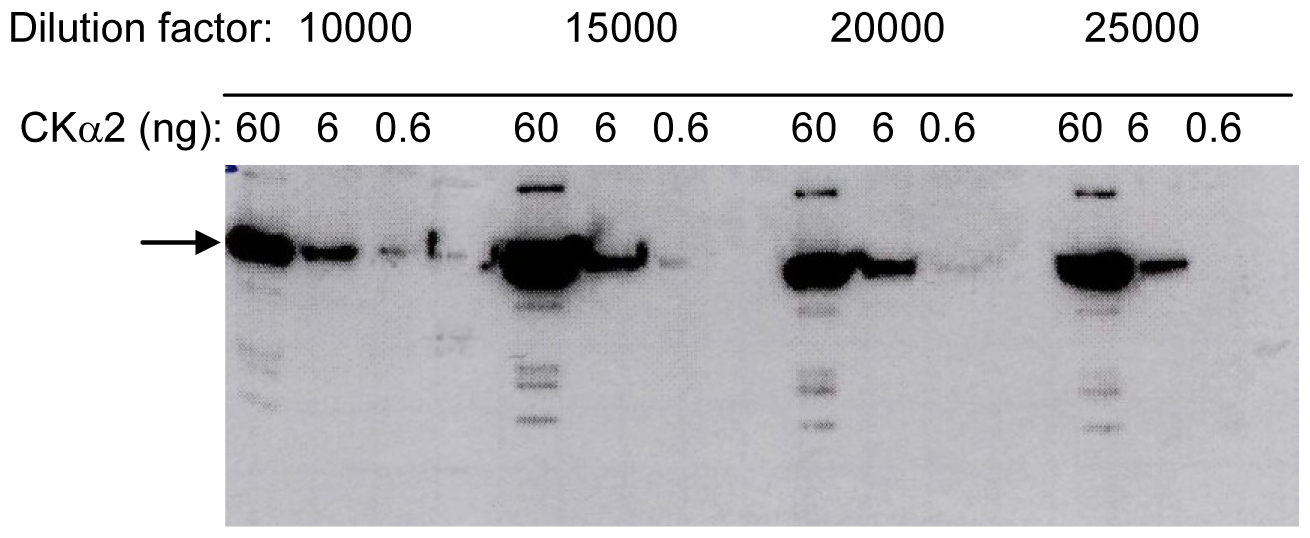
Immunoblot detection of purified CKα2 showing high sensitivity of CKα antiserum. Varying amounts of purified human CKα2 were detected using different dilutions of CKα antiserum. 6 ng of CKα2 was still detectable with 25000-fold dilution of the antiserum. Arrow indicates the location of hCKα2.

To establish an effective working concentration, different dilutions of the antiserum were used for the detection of purified human CKα2 protein. Figure 1 shows the Western blot detection of three different quantities (60, 6 and 0.6 ng) of CKα2 with four different dilutions (1.0, 1.5, 2.0 and 2.5×10^4^ times) of antiserum. The results indicate that 25000-fold dilution of the antiserum (maximum dilution used in this experiment) was still sufficient to detect the 6 ng band. The lowest CKα2 quantity used (0.6 ng) was detectable by the 20000-fold dilution of the antiserum. However, the signal of the 0.6 ng band was not much different when higher concentrations of the antiserum were used. The results showed that the antiserum was able to detect CKα2 protein at such low concentration.

**Figure 2:**
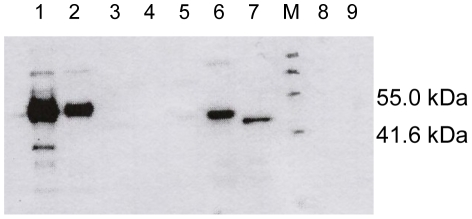
Immunoblot detection of human and baker's yeast choline and ethanolamine kinases showing isoform specificity of CKα antiserum. Detection of purified hCKα1 (1), hCKα2 (2), hCKβ (3), Δ89N-hEK1 (4), hEK2α (5), Δ49N-hCKα2 (6), Δ84N-hCKα2 (7), yCK (8) and yEK (9) were performed with 10000-fold dilution of CKα antiserum. Each lane was loaded with 50 ng of purified protein. Lane M is ChemiBlot molecular weight marker.

To investigate the specificity of the CKα antiserum against proteins in the CK/EK family, it was tested for cross-reactivity with multiple purified human and yeast CK/EK variants (as listed in Table 1). The results in Figure 2 show that the antiserum was very specific for CKα derivatives (hCKα1, Δ49N- and Δ84N-hCKα2). The N-terminal truncation of CKα2 significantly reduced the signal produced by the antiserum. No cross reactivity with purified hCKβ, Δ89N-hEK1, hEK2α, yCK, and yEK was detected.

**Table 1:** Proteins and expression plasmids used in this study.

Protein [reference]	Expression plasmid	Molecular weight (kDa)
Human CKα1 [5]	pGEX-RB-hCKα1	50.1
Human CKα2 [5]	pGEX-RB-hCKα2	52.2
Human CKβ [5]	pGEX-RB-hCKβ	45.3
Human CKα2 truncated at the first 49 amino acids [22]	pGEX-RB-Δ49N-hCKα2	47.5
Human CKα2 truncated at the first 84 amino acids (produced in this work)	pGEX-RB-Δ84N-hCKα2	43.9
Human ethanolamine kinase 1 (EK1) truncated at the first 89 amino acids (produced in this work based on [34])	pET-14b-Δ89N-hEK1	42.0
Human EK2α [34]	pET-14b-hEK2α	44.8
*Saccharomyces cerevisiae* CK [35]	pGEX-RB-yCK	66.3
*Saccharomyces cerevisiae* EK [36]	pGEX-RB-yEK	61.7

pGEX-RB and pET-14b were used for expression as GST and 6x histidine fusion proteins, respectively.

**Figure 3:**
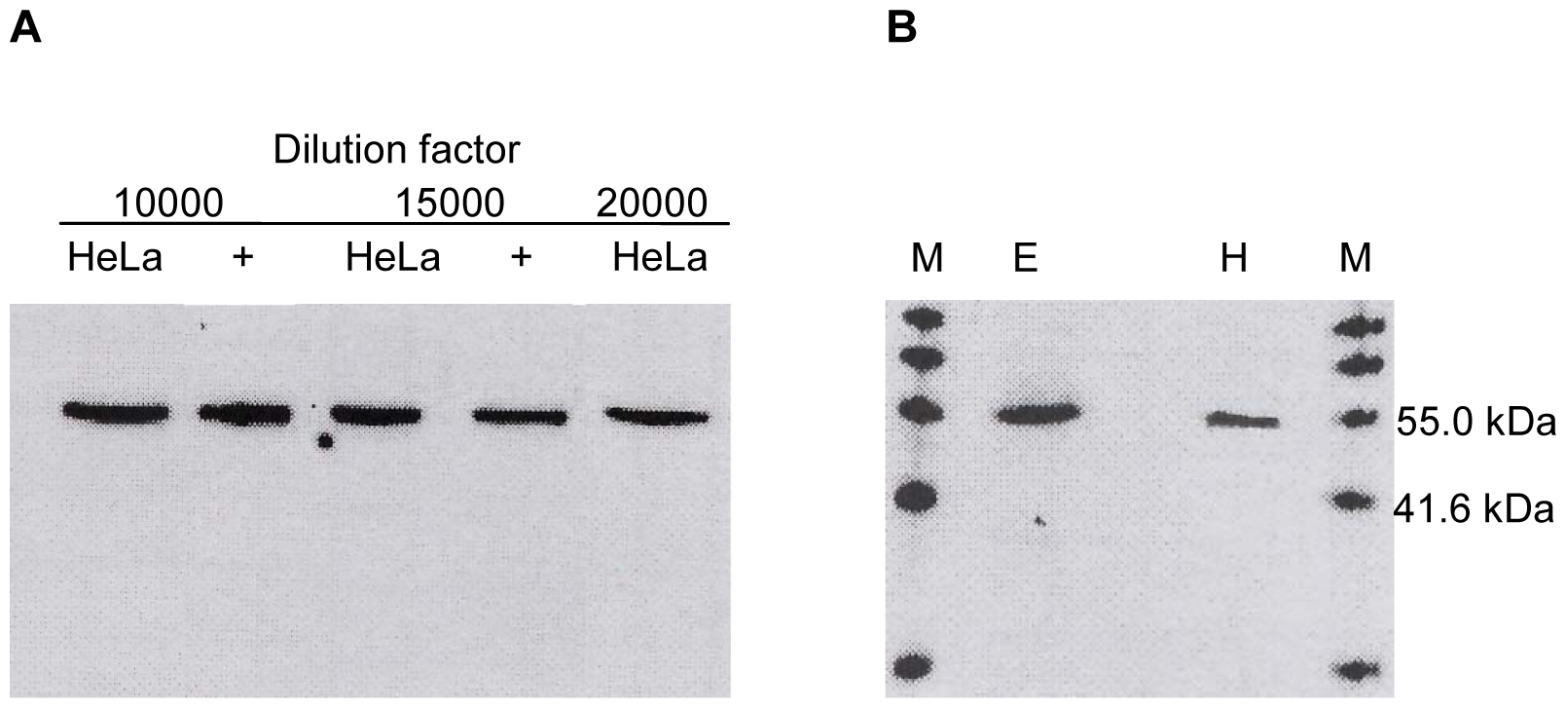
Immunoblot detection showing specificity of CKα antiserum in HeLa (A) and mouse embryonic stem cell (B) lysates. The detection was performed with different dilutions of CKα antiserum for HeLa cell lysate. 6 ng of purified hCKα2 was used as the positive control (+). 10000-fold dilution of CKα antiserum was used for detection of mouse CKα in 50 µg of mouse embryonic stem cell protein lysate (E), using 50 µg of HeLa cell protein extract as the positive control (H). M: ChemiBlot molecular weight marker. Results are representative of triplicate experiments with similar results.

The CKα antiserum was subsequently tested with HeLa cell extract. As shown in Figure 3A, CKα was detected with up to 20,000-fold dilution, and there was no apparent unspecific detection of other proteins including human CKβ, EK1 and EK2, or other unknown human choline or ethanolamine kinases. Only a single band was detected in HeLa cell extract although both CKα1 and α2 could be detected by this antiserum. The band detected in HeLa cell extract corresponded exactly to the positive control, which was 6 ng of purified human CKα2, suggesting that only this isoform was present in HeLa cells.

### CKα antiserum also detects mouse choline kinase

CKα antiserum was tested with mouse embryonic stem cell extract. The antiserum was also able to detect mouse choline kinase without any unspecific detection as indicated by a single band in Figure 3B. The size of the protein detected is in agreement with the 49.9 kDa mouse choline kinase α1 isoform [5]. Blast search in the GenBank database revealed that the mouse choline kinase α1 isoform (accession number BAA88153) is highly similar to the human CKα1 isoform, with a protein sequence identity of 88%.

### CKα antiserum detects both α1 and α2 isoforms in certain cancer cell lines

**Figure 4:**
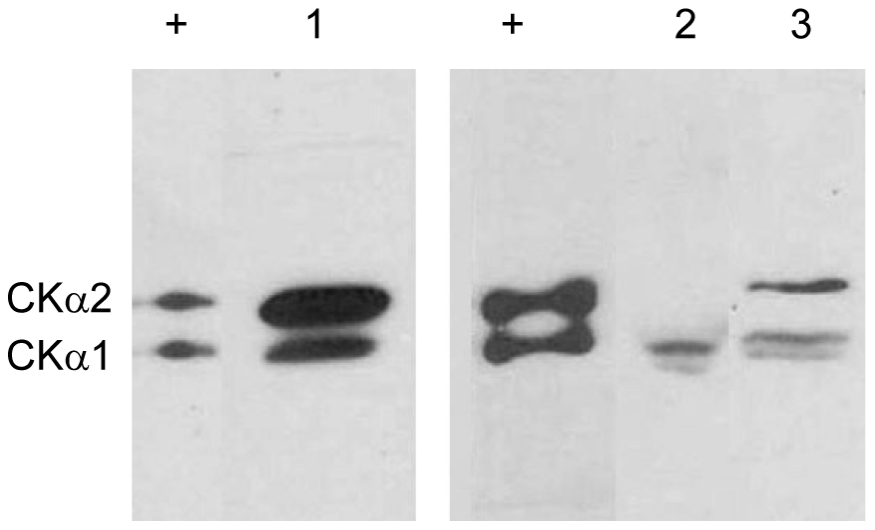
Concurrent immunoblot detection of CKα1 and α2 isoforms in MCF-7 (lane 1) and HepG2 (lane 3) cell lysates. Only CKα1 was detected in the HCT-116 cell lysate (lane 2). 5 ng of each purified CKα1 and α2 were loaded as references (lane +). 50 µg of each cell lysate were loaded and detection was performed with 10000-fold dilution of CKα antiserum. Results are representative of triplicate experiments with similar results.

The presence of CKα was analyzed in three other human cancer cell lines, namely MCF-7 (breast cancer), HCT-116 (colon cancer) and HepG2 (liver cancer). Interestingly, both CKα1 and α2 were detected in MCF-7 and HepG2 cell lysates (Figure 4). The purified CKα1 and α2 enzymes, that were included as reference, were clearly separated and the antiserum showed equal affinity towards both isoforms as evidenced by the equal intensity of signals. Based on its migration on the gel, the band detected in HCT-116 cell lysate was likely to be CKα1. MCF-7 cell line showed higher expression of total CKα compared to HCT-116 and HepG2 cells. The levels of CKα1 and α2 were very similar in cells that expressed both isoforms. All the results presented above showed that the antiserum was sensitive and specific for the detection of CKα isoforms without requiring any purification.

### CKα is differentially expressed in multiple human normal and tumor tissues

**Figure 5:**
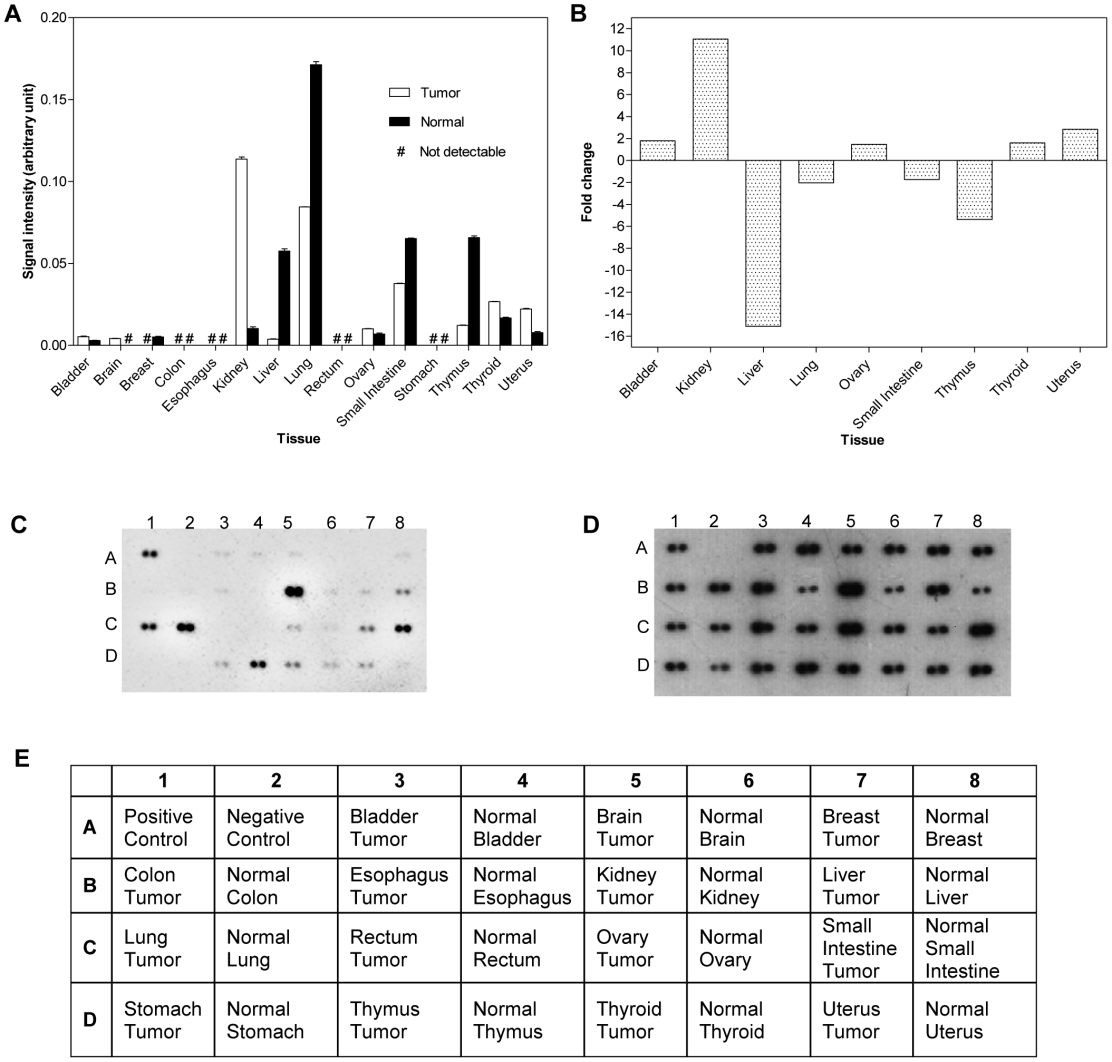
Differential expression of CKα in human normal and tumor tissues. (A) Levels of total CKα in 30 human normal and tumor tissues. The signal intensities (arbitrary unit) were normalized with respect to the corresponding GAPDH signals. (B) Pairwise comparison of tumor/normal CKα levels. Only tissues showing detectable levels of CKα in both tumor and normal samples were analyzed. Positive and negative fold changes indicate higher and lower expression in tumor, respectively. (C) CKα signal detected on the panel by using 20000-fold diluted CKα antiserum. (D) GAPDH detection assay provided by the supplier; it was used for signal normalization. (E) Identities of protein spots on the panel.

The specificity of the CKα antiserum when tested on a series of human cell lysates and mouse embryonic stem cell extract supported its use in non-homogeneous samples such as for protein dot blot analysis of total CKα. In this study, a commercial protein array consisting of 30 different protein samples from human normal and tumor tissues was used for detection of total CKα by using the CKα antiserum. Stripping and re-probing of the array produced identical results albeit with lower signal intensity. CKα expression was detected in 70% of the tissue samples on the panel, and the detected signal intensities of different tissues after normalization with respect to the corresponding GAPDH signals are presented in Figure 5A. The expression levels of CKα in different human normal and tumor tissues displayed a very large range with higher levels of CKα expression in normal lung and kidney tumor tissues followed by lung tumor, normal small intestine, normal thymus and normal liver tissues.

Tissues with detectable levels of CKα in both tumor and normal samples were subjected to pairwise comparison. Figure 5B shows that about half of the tissues compared showed significantly higher expression of CKα in tumors. The most significant differences between the level of CKα in tumor and normal tissues were observed in kidney with 11-fold higher expression in tumor, whereas in liver, 15-fold higher expression was found in normal samples. According to the information provided by the manufacturer of the protein array, the kidney tumor sample was a well differentiated clear cell carcinoma from a 44 year old female.

## Discussion

Antibody against human CKα was raised in rabbits using purified full length human CKα2 protein as the immunogen. The antiserum showed high sensitivity and specificity for CKα1 and α2 isoforms, without purification of the IgG fraction, as evidenced by its ability to detect minute amount of CKα protein, with no apparent cross-reactivity with other human or yeast CK and EK isoforms. Previous antibodies against human choline kinase were generated by immunizations with either GST-purified full-length CKα protein [17], [18] or synthetic peptide [10]. However, all the antibodies, either monoclonal or polyclonal, detected either CKα1 or α2 in the same sample. The first antibody raised against choline kinase was a polyclonal antibody against rat CKα [7]. This antibody detected choline kinase α1 and, to a lesser extent, α2 in colon cancer tissues of rats treated with 1,2 dimethylhydrazine [20]. However, the same antibody only detected a faint 52 kDa protein that corresponded to the size of CKα2 in human normal and cancer colon cells [21]. In this report, we showed that purified CKα1 and α2 were separable in standard SDS-PAGE, and these purified proteins could serve as a reference for simultaneous detection of the two isoforms in cell lysates. Interestingly, only two of the four cell lines tested in this work expressed detectable level of both isoforms. It remains to be determined whether the expression of CKα1 and α2 isoforms is cell cycle or cell type dependent, or whether it is also governed by other factors such as growth factors and carcinogens.

Although RT-PCR or quantitative real-time PCR have been used to assess the level of CK expression [14], it is not possible to design specific primers for human CKα1 because its whole cDNA sequence is shared with the sequence of CKα2. The unique properties of CKα antiserum combined with the purified CKα1 and α2 proteins as references, will be an attractive tool for accurate quantification and comparison of total or individual CKα isoforms in various cells, especially because the antiserum also showed identical affinity towards both isoforms in our experiments. We have previously shown that human CKα1 and α2 have very distinct catalytic activities, the α2 isoform being about four times more active than α1, with much higher affinity for choline [22]. Therefore, the differential detection and quantification of the two isoforms is of critical importance for the design of a more specific inhibitor that targets only the elevated isoform.

The specificity of CKα antiserum also makes it suitable for immunoprecipitation of total CKα from cell extract to study the *in vivo* properties of this isoform in terms of post-translational modifications and interaction with other CK isoforms [23] or proteins. Recently, RNA interference has been successfully employed to selectively knockdown different isoforms of CK [10], [15], [24]. The antiserum can provide confirmation of CKα silencing in such experiments. Moreover, reactivity of the CKα antiserum with mouse choline kinase will allow for broader applications of this antiserum as mouse is widely used as an animal model for pharmacological, developmental and immunological studies [25], [26].

Total CKα was detectable in two thirds of the tissue samples displayed on the protein array. This observation is in agreement with Northern blot analyses of CKα mRNA levels in human, mouse, and rat that showed it was ubiquitously expressed in various tissues of the three mammalian species [23]. Overexpression of choline kinase has been reported to be a frequent feature in human tumor-derived cell lines and in lung, prostate and colorectal cancers [6]. On the contrary, the results from our protein dot blot assay showed that overexpression of CKα was not observed in all tumor tissues where only about 50% of tumor samples expressed higher levels of CKα compared to their normal tissues. These results are, however, explainable since only 17%, 47%, 56% and 48% of breast, colon, lung and prostate cancers, respectively, showed increased CKα levels [6], [13]. The implication of choline kinase in human cancer pathogenesis was not entirely due to a higher expression level of this enzyme, but also to its higher activity detected in cancer cells [13]. The choline kinase β isoform, which is not detected by the CKα antiserum, or the more active CKα2 alone could also contribute to the higher choline kinase activity in cancer cells [21]. The higher choline kinase activity can also result from post-translational activation such as phosphorylation or positive regulation by interacting partners.

The use of protein array enabled us to analyze CKα levels in a relatively large number of tissue samples. The array is particularly applicable with the antiserum produced in this work since Western blots did not show any cross reactivity with other human proteins, and therefore the signal from the protein dot blot should reliably reflect the level of total CKα. The use of the protein array also bypassed the time-consuming sample collection step especially for tissue samples from human volunteers. The protein array screening strategy proved to be highly suitable for identifying tissues with detectable and significantly different levels of CKα in normal and tumor samples. Results obtained with the protein panel could be confirmed by Western blot analysis of more samples from the tissue of interest.

We note that the protein array may have disadvantages, such as the limited sample number (only one) for each type of tissue, which greatly affects the statistical reliability of any observation, and the loss of signal intensity after stripping and re-probing, which limits the use of the array for further probing with other antibodies. Use of the CKα antibody can be extended to protein microarrays that cover more tissue types and samples [27], [28]. Our antiserum reduces the risk of unspecific detection in non-homogenous samples, and it can be used to determine the level of CKα isoforms in different types and stages of cancer, as well as in various samples such as blood plasma.

Based on the emerging evidences that specifically link CKα to the pathogenesis and prognosis of various cancers [15], [16], [29], [30], it is apparent that more focus should be given to the detection of this isoform. Our antibody provides the opportunity to study the regulation of both CKα1 and α2 expression in different cells and tissues through direct quantitative comparison of the expressed protein pattern. More specifically, the antibody could potentially be used to monitor the CKα expression during different stages of cancer development and subsequently for monitoring tumor response to treatments.

## Materials and Methods

### Expression and purification of human and yeast choline/ethanolamine kinases

The cDNA sequences coding for the proteins of interest were ligated into the *Nde*I and *BamH*I sites of pET-14b (Novagen) or pGEX-RB [31] vectors for expression as 6x histidine or glutathione S-transferase (GST) fusion proteins, respectively. The proteins and their expression plasmids used in this study, together with the predicted molecular masses of the CK subunits are listed in Table 1. All proteins were expressed in *Escherichia coli* C41(DE3) strain [32]. The culture was first grown at 37°C until OD_600 nm_ of 0.8 to 1.0 was reached. Protein expression was induced by 0.3 to 1.0 mM (final concentration) of IPTG at 25°C for 16 hours. Subsequently, the cells were harvested by centrifugation at 5000×g for 20 minutes at 4°C. The cell pellet was re-suspended in pre-chilled (4°C) lysis buffer containing 50 mM Tris-HCl (pH 7.5), 300 mM NaCl, 5 mM EDTA, 10% glycerol, 1% Triton X-100, 5 mM β-mercaptoethanol, 0.5 mM phenylmethylsulphonylfluoride (PMSF) and 1 tablet of Complete™ protease inhibitor cocktail (Roche) in every 50 ml of buffer. The lysis buffer used for purification of His-tag protein was supplemented with 10 mM imidazole to reduce non-specific binding. The cell suspension was sonicated in an ice bath with short pulses of 1 second burst and 1 second pause for 1 to 4 minutes. After the sonication step, the cell lysate was centrifuged at 15000×g for 30 minutes (4°C) and the supernatant was transferred into a new tube. The His-tagged and GST-tagged proteins were purified under native conditions by affinity binding to nickel-nitriloacetic acid (Ni-NTA) metal affinity matrix (Qiagen), and Glutathione Sepharose 4B (GE Healthcare), respectively. The matrix was washed with 20 bed volumes of wash buffer (50 mM Tris-HCl (pH 7.5), 300 mM NaCl, 0.5% Triton X-100 and 10% (v/v) glycerol). Proteins were eluted without the affinity tags by overnight cleavage with thrombin (Serva Electrophoresis). Protein concentration was determined by the Bradford assay, and enzymes were stored at −80°C. All purification steps were carried out at 4°C in a cold room.

### Gel purification of antigen and antibody production

Affinity purified human CKα2 was run on a preparative 12% SDS-PAGE gel at 70 mA for 3 hours in the cold room. After Coomassie staining, the protein band corresponding to the size of CKα2 was excised, sliced into smaller pieces (about 0.25 cm×2 cm), and eluted from the gel using the Elutrap electroelution system (Schleicher and Schuell) at 30 mA for 16 hours at 4°C with 1x TAE buffer. For production of polyclonal CKα antibodies in rabbits, 1 mg of the eluted protein was used as immunogen (Eurogentec, Belgium). The standard immunization program recommended by the company was followed without modification. The serum from the final bleeding (87 days after first immunization) was used as antiserum for Western blot detection, without isolation of the IgG fraction.

### Cell cultures and preparation of cell lysates

Human HeLa (ATCC no. CCL-2), MCF-7 (ATCC no. HTB-22), HCT-116 (ATCC no. CCL-247) and HepG2 (ATCC no. HB-8065) cell lines were maintained in Dulbecco's Modified Eagle Medium (DMEM) with 10% (v/v) fetal calf serum and supplemented with 100 U/ml of penicillin/streptomycin antibiotic mix (Sigma), under standard conditions of 37°C and 5% CO_2_. To prepare the cell lysates for Western blotting, culture medium was removed and cells were washed once with PBS buffer. Protein was extracted by Proteojet mammalian cell lysis buffer (Fermentas) and quantified by the Bradford assay (Biorad). Mouse embryonic stem cell extract was provided by Dr. Luo Ling Fei from the Department of Developmental Biology, MPI-BPC, Germany.

### Western blot, sensitivity and specificity analyses of CKα2 antiserum

Variable amounts of purified proteins or 50 µg of cell lysates were separated on 12% SDS-PAGE and transferred onto nitrocellulose membrane by using the ‘tank blot’ method [33]. The gel was placed on a nitrocellulose membrane (Schleicher and Schuell) of equal size and sandwiched with 2 layers of Whatman paper. The stack was transferred into the blot chamber (BioRad Trans-Blot cells) filled with transfer buffer (20 mM Tris-base, 150 mM glycine, 20% (v/v) methanol) and electroblotted at 110 mA for 1.5 to 2 hours at 4°C. Successful protein transfer was indicated by the blotting of pre-stained protein ladder (Invitrogen).

After the transfer step, the membrane was immersed in blocking solution [Western buffer A (10 mM Tris-HCl (pH 7.5), 150 mM NaCl, 0.1% (v/v) Tween 20), supplemented with 2% (w/v) milk powder) and shaken gently for 1 hour at room temperature. After removal of the blocking solution, the membrane was incubated with a fresh blocking solution containing the primary antibody at a specific dilution (1∶10000 to 1∶25000) for 1 hour. The membrane was subsequently washed 3 times for 10 minutes with Western buffer A followed by incubation with donkey anti-rabbit IgG (Amersham) secondary antibody (dilution 1∶5000) in blocking solution for 45 minutes. The membrane was washed 6 times for 10 minutes with Western buffer A before standard enhanced chemiluminescence (ECL) detection whereby the membrane was incubated for one minute in a 1∶1 mixture of the two ECL solutions (Roche) and immediately analyzed on the Lumi-Imager workstation (Boehringer).

### Protein dot blot detection of CKα in human normal/tumor tissue panel

Human normal/tumor protein array purchased from BioChain Institute (catalog no. A1235712; lot no. A605122) was probed with CKα antiserum at 1∶20000 dilution according to the standard Western detection protocol as described above. Based on the information provided by the manufacturer, the protein array was made by spotting the same amount of total protein from 15 pairs of human normal and tumor tissues on a 1″×2″ nylon membrane. The protein array included two positive (normal placenta) and two negative (water) control spots. All samples were spotted in duplicate. The characteristics and donor information of protein samples on the panel were provided by the manufacturer. Western detection of control protein, glyceraldehyde 3-phosphate dehydrogenase (GAPDH), on the same batch of protein array was carried out by the manufacturer and the result was provided together with the product. Signals detected on the panels were analyzed by using ImageJ version 1.43 U software (downloaded from http://rsbweb.nih.gov/ij/). Briefly, the background was subtracted with rolling radius of 50 pixels under light background selection and the color of the image was inverted. The integrated densities of dots representing each tissue were measured in triplicate and the average values were then normalized to the corresponding integrated densities of GAPDH. Detectable limit was defined as the signal intensities that were at least three times higher than the signal intensity of background. All values were expressed as means ± SD. Statistical analysis was performed with GraphPad Prism 5. CKα expressions in normal and tumor tissues were compared using the one-way analysis of variance (ANOVA). *P*-values <0.05 were considered as statistically significant.
